# Fast Driving of a Particle in Two Dimensions without Final Excitation

**DOI:** 10.3390/e24111694

**Published:** 2022-11-19

**Authors:** Xiao-Jing Lu, Mikel Palmero, Ion Lizuain, Juan Gonzalo Muga

**Affiliations:** 1 School of Science, Xuchang University, Xuchang 461000, China; 2 Departamento de Química Física, University of the Basque Country UPV/EHU, Apdo. 644, 48080 Bilbao, Spain; 3 Department of Applied Physics, University of the Basque Country UPV/EHU, 48013 Bilbao, Spain; 4 EHU Quantum Center, University of the Basque Country UPV/EHU, 48940 Leioa, Spain; 5 Department of Applied Mathematics, University of the Basque Country UPV/EHU, Plaza Europa 1, 20018 Donostia-San Sebastián, Spain

**Keywords:** shortcuts to adiabaticity, trap rotations, linear invariants

## Abstract

Controlling the motional state of a particle in a multidimensional space is key for fundamental science and quantum technologies. Applying a recently found two-dimensional invariant combined with linear invariants, we propose protocols to drive a particle in two dimensions so that the final harmonic trap is translated and rotated with respect to the initial one. These protocols realize a one-to-one mapping between initial and final eigenstates at some predetermined time and avoid any final excitations.

## 1. Introduction

Coupled harmonic oscillators are key models in physics as they describe many different systems near equilibrium. In particular, they play a fundamental role in several quantum technologies such as simulation, communication, and information processing [1,2,3,4,5,6]. Inverse engineering the dynamics of two coupled oscillators is thus a basic and important operation for controlling quantum systems. In a previous publication [7] we demonstrated that when the coupling is proportional to the product of oscillator coordinates xy, it is possible to inverse engineer the time dependence of the quadratic potential using a combination of invariants to swap the quantum numbers of any eigenstate of the initial uncoupled oscillators. The process may be faster than the adiabatic one and it is not state-specific, in other words, the initial quantum numbers need not be known. In more detail, we identified first a “family” of driving processes based on a quadratic invariant found by Simsek and Mintert [8,9,10]. This invariant is degenerate except for the ground state, so the processes in this family do not guarantee swapping in general. To remedy the “degeneracy problem” we complemented the quadratic invariant with linear invariants and found an explicit expression for the final states, which may become the desired swapped states by adjusting the value of a single control parameter (once and for all for a predetermined process time, i.e., for arbitrary quantum numbers). The adjustment was done with very little numerical effort by running classical trajectories until a boundary condition is met.

The specific application we worked out in ref. [7] was the swapping of quantum numbers describing a single particle state in a two-dimensional harmonic trap whose final configuration is rotated by π/2 with respect to the initial configuration (The intermediate driving though is not a pure trap rotation since the eigenfrequencies are also deformed along the process). Up to a phase factor, which may be manipulated, the final state was a replica, rotated by π/2, of the initial eigenstate. On a rotating basis, the protocol in [7] facilitates a one-to-one mapping between initial and final eigenstates keeping at the final time the same quantum numbers set initially. In other words, the “swapping” results from defining the quantum numbers instead in non-rotating bases for the initial and final oscillators in *x* and *y* directions, which were the principal axes directions of the initial and final trap configurations. We shall generalize these results to allow for more general trap manipulations in 2D including arbitrary rotations and displacements. These extensions provide a theoretical background for shuttling operations in 2D networks using trapped ions in multisegmented Paul traps [11] or neutral atoms driven by optical tweezers [12], but we shall leave aside, except for some comments in the final discussion, implementation issues, which should be studied separately and vary largely with the setting and system. The first generalization considered here is to allow for rotations by an arbitrary final angle $\theta_{f}$ (in the examples we use π/3). The particular, the π/2 angle treated in [7] is somewhat simpler, because the directions of the initial and final principal axes coincide, and ref. [7] only hinted at how to deal with other angles. Moreover, ref. [7] made use of a restricted subset of the Hamiltonians allowed in ref. [8], whereas in this paper we shall make use of the whole set to combine trap rotations and displacements of the eigenstates of the initially uncoupled oscillator map, one to one, with the final eigenstates of the final oscillators.

The work in ref. [7], allowing for a fast, controlled rotation of the final trap with respect to the initial trap, together with known shortcut-to-adiabaticity (STA) [13] protocols for 1D displacements and trap expansions or compressions, form in principle a complete toolbox of elementary motional drivings in 2D. These elementary processes could drive sequentially (i.e. by means of a succession of translations and a rotation) an arbitrary 2D trap on a plane to any other trap (with different locations, orientations, and eigenfrequencies), implementing a one-to-one mapping from initial to final eigenstates. However, sequential processes may be outperformed by combined ones in which the different operations are done simultaneously [14], for example, to turn a corner. We shall design such combined protocols here.

**Figure 1:**
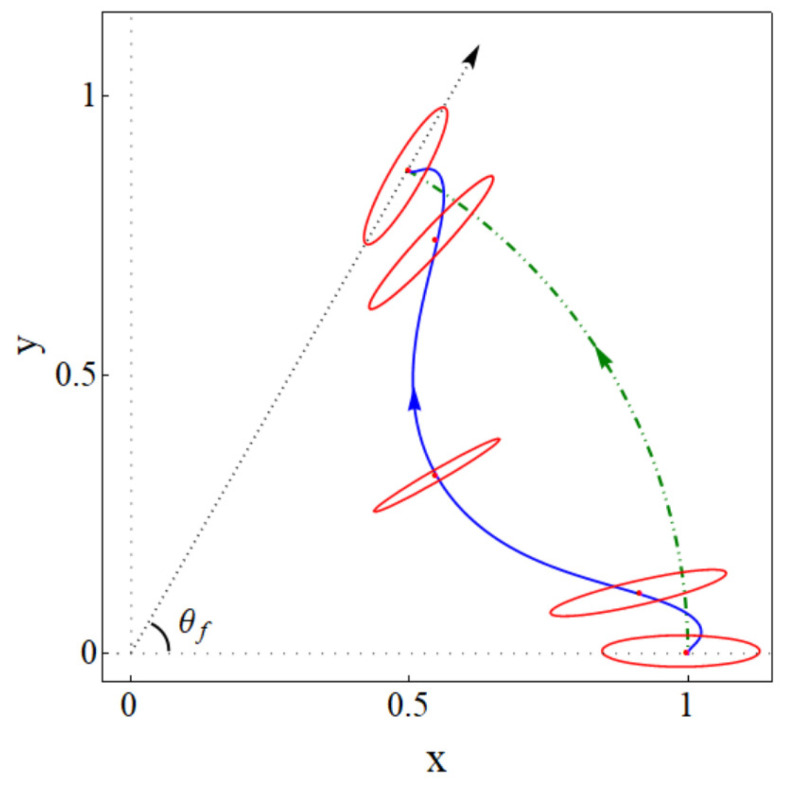
(Color online). Schematic representation of initial (long principal axis in *x* direction), intermediate, and final configurations (long principal axes along the $\theta_{f}$ radius) of a driving 2D harmonic trap (red ellipses). The trap trajectory (the blue solid line is the trajectory of the trap center) drives any initial eigenstate along the green dotted-dashed line to become a corresponding (rotated and translated) final eigenstate. The ellipses are equipotential lines from the trap bottom drawn at equal time intervals.

Figure 1 depicts an example of a designed state trajectory as well as the inverse engineered trap trajectory found in this work to drive that desired state evolution. We also depict schematic snapshots (ellipses) of the evolution of the trap at equal time intervals.

**Figure 2:**
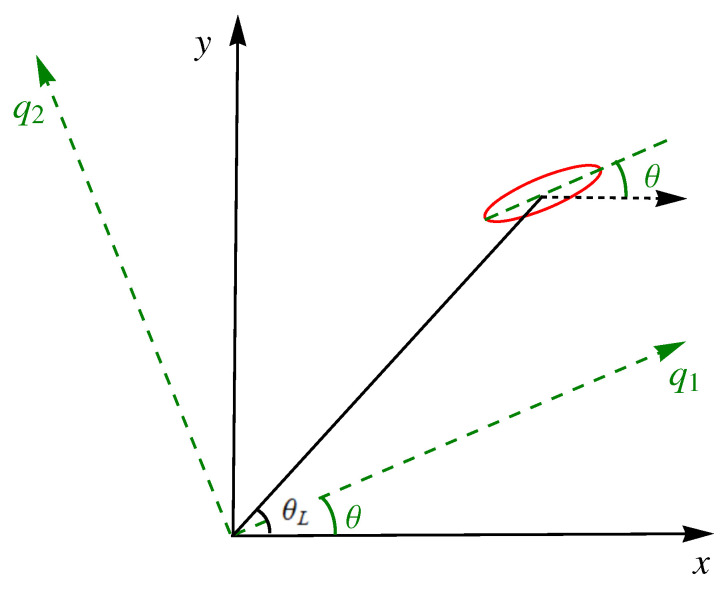
(Color online). Schematic representation of a harmonic trap (red ellipse) centered at some point in the x,y plane. The principal axes of the trap are rotated with respect to the *x* and *y* directions by an angle θ. This rotation angle defines the auxiliary rotating frame $q_{1},q_{2}$ with the same origin as the (fixed, or “laboratory”) x,y axes. The polar angle for the center of the trap is $\theta_{L}$. In general, $\theta \neq \theta_{L}$ although they coincide at initial and final times ($\theta \left(0\right)=\theta_{L}\left(0\right)=0,\theta \left(t_{f}\right)=\theta_{L}\left(t_{f}\right)=\theta_{f}$) in the process represented in Figure 1.

A clarification on notation and terminology: “trap rotation” is understood here as a rotation of the principal axes of the trap with respect to fixed directions in the laboratory frame (the rotation may occur simultaneously with deformations of the principal frequencies and translations). The rotation is characterized by an angle θ(t) that is also used to define rotating coordinates $q_{1},q_{2}$ parallel to the principal axes but having the same origin as the x,y axes, see Figure 2. We also define a polar angle for the center of the trap $\theta_{L}\left(t\right)$ in the (x,y) lab frame, see Figure 2. These two angles are, in general, different, but in numerical examples below and in Figure 1, they are made equal at initial and final times, $\theta \left(0\right)=\theta_{L}\left(0\right)=0,\theta \left(t_{f}\right)=\theta_{L}\left(t_{f}\right)=\theta_{f}$. This coincidence at boundary times is a natural choice for possible applications but it is not a necessary condition for the applicability of the inverse method worked out in the following.

The paper is organized as follows: in Section 2, we describe the two-dimensional invariant adapted from Simsek et al. [8]; in Section 3, we set the model and notation; Section 4 gives the ansatzes for inverse engineering, and in Section 5 the degeneracy of the invariant is discussed; in Section 6, auxiliary linear invariants are introduced to solve the degeneracy problem. The article ends with a discussion and technical appendices.

## 2. A Quadratic Invariant for Two-Dimensional Harmonic Traps

Simsek and Mintert [8] consider Hermitian Hamiltonians with a 4×4 matrix form

(1) $$\begin{array}{c} H=\frac{1}{2}{\hat{X}}^{T}\Omega \hat{X}+{\hat{X}}^{T}V,\;\;\;\\ \Omega =\left(\begin{array}{cc} M & 0\\ 0 & 1 \end{array}\right),V=\left(\begin{array}{c} -F\\ 0 \end{array}\right), \end{array}$$

where ${\hat{X}}^{T}=\left(x,y,p_{x},p_{y}\right)$, with $p_{x}$ and $p_{y}$ being conjugate momenta for *x* and *y*, 1 represents a 2×2 unit matrix, and the superscript *T* means the transpose of the matrix. The formal treatment is dimensionless here (see a discussion of the dimensionless units in ref. [7]) so that no mass or *ℏ* appear explicitly in any equation. As a reminder, the unit of (angular) frequency is one of the initial eigenfrequencies of the trap, and the unit of time its inverse. The potential has a quadratic part with real coefficients in the time-dependent 2×2 symmetric matrix *M*, and a linear part characterized by the force vector *F*,

(2) $$\begin{array}{c} M=\left(\begin{array}{cc} M_{xx} & M_{xy}\\ M_{yx} & M_{yy} \end{array}\right)\;,\;F=\left(\begin{array}{c} F_{x}\\ F_{y} \end{array}\right). \end{array}$$

Here *M* determines the eigenfrequencies and orientation of the “trap”, which may be as well a saddle, or an antitrap if one or two eigenfrequencies become imaginary (When we refer to a “trap” from now on, these possibilities—a saddle or an antitrap—should also be included), whereas *F* determines its displacement from the origin (The exact displacement is in Equation (19) below). Simsek and Mintert find quadratic invariants compatible with this Hamiltonian of the form [8]

(3) $$\begin{array}{c} I_{0}\left(t\right)=\frac{1}{2}{\hat{X}}^{T}\Gamma \hat{X}+{\hat{X}}^{T}W+\gamma , \end{array}$$

where Γ ($\Gamma^{-1}/2$ is the covariance matrix of the Gaussian ground state of the invariant) is given by

(4) $$\begin{array}{c} \Gamma \;=\;\left(\begin{array}{cc} m({\dot{R}}^{2}+Re\left({\left[\dot{R},R\right]}_{A}-RA^{2}R\right)) & \frac{J-\{R,\dot{R}\}}{2}\\ \frac{-J-\{R,\dot{R}\}}{2} & \frac{R^{2}}{m} \end{array}\right), \end{array}$$

and the dots represent time derivatives. *W* and γ are given by

(5) $$W=-\Gamma \left(\begin{array}{c} L\\ \dot{L} \end{array}\right),\;\;\;\gamma =\frac{1}{2}W^{T}\Gamma^{-1}W,$$

where *R* is a real, Hermitian, positive semidefinite 2×2 matrix, and *L* is a real vector, satisfying

(6) $$\begin{array}{ccc} \left\{\ddot{R},R\right\}+\left\{R^{2},M\right\} & = & 2{\left[\dot{R},R\right]}_{A}-2RA^{2}R, \end{array}$$

(7) $$\begin{array}{ccc} \ddot{L}+ML & = & F, \end{array}$$

with *A* and J determined from

(8) $$\begin{array}{ccc} A & = & iR^{-2}+\frac{1}{2}\left[R^{-1},\dot{R}\right]+\frac{1}{2}R^{-1}JR^{-1},\\ \left\{J,R^{-2}\right\} & = & \left[\dot{R},R^{-1}\right]+{\left[R,R^{-2}\right]}_{\dot{R}}. \end{array}$$

Here ${\left[X,Y\right]}_{Z}=XZY-YZX$ and {X,Y}=XY+YX is the anti-commutator.

Equation (6) is a generalized (matrix) Ermakov equation. When inverse-engineering the Hamiltonian, the designed *R* determines the form of the quadratic part *M*. $R^{2}/2$ is physically the spatial correlation matrix for the Gaussian ground state of the invariant. In the (slow) adiabatic limit, $R\to M^{-1/4}$. Equation (7) has the form of a vector Newton equation. *L* gives the trajectory of the center of the ground state of the invariant [8]. Using this equation inversely, the designed (state) trajectory *L* together with *M* will determine the homogeneous two-component force *F*.

*R* and *L* are designed by setting their boundary conditions and interpolating between them. Analogously to one dimensional (1D) configurations [15,16], the boundary conditions are chosen so that

(9) $$\left[I_{0}\left(0\right),H\left(0\right)\right]=\left[I_{0}\left(t_{f}\right),H\left(t_{f}\right)\right]=0,$$

which implies

(10) $$\begin{array}{ccc} \ddot{R}\left(t_{b}\right) & = & \dot{R}\left(t_{b}\right)=0,\;\;\;R\left(t_{b}\right)=M{\left(t_{b}\right)}^{-\frac{1}{4}}, \end{array}$$

(11) $$\begin{array}{ccc} \ddot{L}\left(t_{b}\right) & = & \dot{L}\left(t_{b}\right)=0,\;\;\;L\left(t_{b}\right)=M{\left(t_{b}\right)}^{-1}F\left(t_{b}\right), \end{array}$$

with $t_{b}=0,t_{f}$ being the boundary times.

## 3. Model for Rotation and Transport

The Hamiltonian (1) takes the explicit form

(12) $$H\left(t\right)=\frac{p_{x}^{2}}{2}+\frac{p_{y}^{2}}{2}+\frac{1}{2}M_{xx}x^{2}+\frac{1}{2}M_{yy}y^{2}+M_{xy}xy-F_{x}x-F_{y}y.$$

It is useful to express *M*, and later the whole Hamiltonian, in terms of rotated coordinates centered at the origin of the laboratory frame, see Figure 2,

(13) $$\begin{array}{c} \left(\begin{array}{c} q_{1}\\ q_{2} \end{array}\right)=U\left(t\right)\left(\begin{array}{c} x\\ y \end{array}\right),\;\;\;\;\left(\begin{array}{c} p_{1}\\ p_{2} \end{array}\right)=U\left(t\right)\left(\begin{array}{c} p_{x}\\ p_{y} \end{array}\right), \end{array}$$

where $p_{1,2}$ are the corresponding momenta and

(14) $$\begin{array}{c} U\left(t\right)=\left(\begin{array}{cc} \cos\theta (t) & \sin\theta (t)\\ -\sin\theta (t) & \cos\theta (t) \end{array}\right). \end{array}$$

The angle θ is chosen so that $q_{1}$ and $q_{2}$ become the natural coordinates along the principal axes for the quadratic part, i.e.,

(15) $$\tan\left(2\theta \right)=\frac{2M_{xy}}{M_{xx}-M_{yy}},$$

with (squared) eigenfrequencies given by

(16) $$\begin{array}{ccc} \omega_{1}^{2} & = & \frac{\cos^{2}\theta }{\cos2\theta }M_{xx}-\frac{\sin^{2}\theta }{\cos2\theta }M_{yy},\\ \omega_{2}^{2} & = & -\frac{\sin^{2}\theta }{\cos2\theta }M_{xx}+\frac{\cos^{2}\theta }{\cos2\theta }M_{yy}. \end{array}$$

The inverse relations are

$$\begin{array}{ccc} M_{xx} & = & \omega_{1}^{2}\cos^{2}\theta +\omega_{2}^{2}\sin^{2}\theta ,\\ M_{yy} & = & \omega_{1}^{2}\sin^{2}\theta +\omega_{2}^{2}\cos^{2}\theta ,\\ M_{xy} & = & M_{xy}=\left(\omega_{1}^{2}-\omega_{2}^{2}\right)\sin\theta \cos\theta . \end{array}$$

In terms of these rotated coordinates and momenta (time-dependent functions of the lab frame coordinates and momenta), the lab-frame Hamiltonian may be rewritten as

(17) $$\begin{array}{ccc} H(t)\; & = & \;\frac{p_{x}^{2}}{2}\;+\;\frac{p_{y}^{2}}{2}\;+\;\frac{1}{2}\omega_{1}^{2}q_{1}^{2}\;+\;\frac{1}{2}\omega_{2}^{2}q_{2}^{2}\;-\;F_{1}q_{1}\;-\;F_{2}q_{2}\\ \; & = & \;\frac{p_{1}^{2}}{2}+\frac{p_{2}^{2}}{2}\;+\;\frac{\omega_{1}^{2}}{2}{\left(q_{1}-\frac{F_{1}}{\omega_{1}^{2}}\right)}^{\;\;2}\;+\;\frac{\omega_{2}^{2}}{2}{\left(q_{2}\;-\;\frac{F_{2}}{\omega_{2}^{2}}\right)}^{\;\;2}\\ & - & \;\frac{F_{1}^{2}}{2\omega_{1}^{2}}-\frac{F_{2}^{2}}{2\omega_{2}^{2}}, \end{array}$$

where

(18) $$\begin{array}{c} \left(\begin{array}{c} F_{1}\\ F_{2} \end{array}\right)=U\left(t\right)\left(\begin{array}{c} F_{x}\\ F_{y} \end{array}\right), \end{array}$$

and, to simplify notation, we have dropped the explicit dependences of $q_{1}\left(x,y;t\right)$, $q_{2}\left(x,y;t\right)$, $p_{1}\left(p_{x},p_{y};t\right)$, $p_{2}\left(p_{x},p_{y};t\right)$, and the dependences on time of $F_{1},F_{2},\omega_{1},\omega_{2}$. The trap-center trajectory, in the lab frame, is given by

(19) $$\begin{array}{c} \left(\begin{array}{c} x_{0}\left(t\right)\\ y_{0}\left(t\right) \end{array}\right)=U^{-1}\left(t\right)\left(\begin{array}{c} F_{1}/\omega_{1}^{2}\\ F_{2}/\omega_{2}^{2} \end{array}\right). \end{array}$$

## 4. Ansatzes for Inverse Engineering

We assume the following initial and final traps

(20) $$\begin{array}{ccc} V_{i} & = & \frac{1}{2}\omega_{1}^{2}\left(0\right){\left(q_{1}-r\right)}^{2}+\frac{1}{2}\omega_{2}^{2}\left(0\right)q_{2}^{2},\\ V_{f} & = & \frac{1}{2}\omega_{1}^{2}\left(t_{f}\right){\left(q_{1}-r\right)}^{2}+\frac{1}{2}\omega_{2}^{2}\left(t_{f}\right)q_{2}^{2}, \end{array}$$

which are depicted in Figure 1. Assuming $w_{1}=\omega_{1}\left(0\right)=\omega_{1}\left(t_{f}\right)$ and $w_{2}=\omega_{2}\left(0\right)=\omega_{2}\left(t_{f}\right)$, the matrix *M* and *F* at initial and final time are

(21) $$\begin{array}{ccc} & & \;M\left(t_{b}\right)\;\;=\;\;\left(\;\;\begin{array}{cc} w_{1}^{2}\cos^{2}\theta_{b}+w_{2}^{2}\sin^{2}\theta_{b} & \left(w_{1}^{2}-w_{2}^{2}\right)\cos\theta_{b}\sin\theta_{b}\\ \left(w_{1}^{2}-w_{2}^{2}\right)\cos\theta_{b}\sin\theta_{b} & w_{2}^{2}\cos^{2}\theta_{b}+w_{1}^{2}\sin^{2}\theta_{b} \end{array}\;\;\right)\;,\\ & & \;F\left(t_{b}\right)\;=\;rw_{1}^{2}\left(\begin{array}{c} \cos\theta_{b}\\ \sin\theta_{b} \end{array}\right), \end{array}$$

where $\theta_{b}=0,\theta_{f}$ are the initial and final angles at the boundary times. At the initial time, U(0) becomes the unit matrix. *L* is chosen as an arch with radius *r*. R(t) and the polar angle $\theta_{L}\left(t\right)$ of the point L(t) are interpolated using polynomials that satisfy the boundary conditions in Equations (10), (11) and (21),

(22) $$\begin{array}{ccc} R(t) & = & p_{r}\left(t\right)R\left(t_{f}\right)\;+\;\left[1-p_{r}\left(t\right)\right]R\left(0\right)+{\left(\frac{t}{t_{f}}\right)}^{3}{\left(1-\frac{t}{t_{f}}\right)}^{3}\;R_{c}, \end{array}$$

(23) $$\begin{array}{ccc} L(t) & = & r\left(\begin{array}{c} \cos(\theta_{f}p\left(t\right))\\ \sin(\theta_{f}p\left(t\right)) \end{array}\right), \end{array}$$

where

(24) $$p\left(t\right)=10{\left(\frac{t}{t_{f}}\right)}^{3}-15{\left(\frac{t}{t_{f}}\right)}^{4}+6{\left(\frac{t}{t_{f}}\right)}^{5}.$$

p(t) is chosen to satisfy p(0)=0, $p(t_{f})=1$, and $\dot{p}\left(0\right)=\ddot{p}\left(0\right)=\dot{p}\left(t_{f}\right)=\ddot{p}\left(t_{f}\right)=0$, whereas $R_{c}$ is a constant matrix [7]

(25) $$\begin{array}{c} R_{c}=\lambda {\left(w_{1}w_{2}\right)}^{-\frac{1}{4}}\left(\begin{array}{cc} 0 & 1\\ 1 & 0 \end{array}\right). \end{array}$$

This choice of $R_{c}$ is done for simplicity after some experimentation [7]. Note that $R_{c}$ is not positive semidefinite, so we numerically check our protocols to make sure that R(t) is. $p_{r}\left(t\right)$ satisfies the same boundary conditions as p(t) but it is chosen as a higher order polynomial in order to have some more freedom in the protocol design,

(26) $$p_{r}\left(t\right)=b_{6}{\left(\frac{t}{t_{f}}\right)}^{6}+\left(10-b_{6}\right){\left(\frac{t}{t_{f}}\right)}^{3}-\left(15-3b_{6}\right){\left(\frac{t}{t_{f}}\right)}^{4}+\left(6-3b_{6}\right){\left(\frac{t}{t_{f}}\right)}^{5}.$$

Without trap translation this additional freedom was not necessary [7], but with trap translation, it will be needed later to solve the “degeneracy problem”. In summary, with the described parameterizations the Hamiltonian is set by choosing the parameters λ and $b_{6}$.

## 5. Degeneracy of the Invariant $I_{0}$

In principle, the Hamiltonian can be inversely designed from the invariant $I_{0}$ described in Section 2 and the boundary conditions in Equations (10) and (11). However, this invariant is degenerate except for the ground state. At the boundary times $t_{b}=0,t_{f}$,

(27) $$\begin{array}{ccc} \Gamma (t_{b}) & = & \left(\begin{array}{cc} M^{1/2}\left(t_{b}\right) & 0\\ 0 & M^{-1/2}\left(t_{b}\right) \end{array}\right),\\ W(t_{b}) & = & -\Gamma \left(\begin{array}{c} L(t_{b})\\ 0 \end{array}\right),\;\;\;\gamma \left(t_{b}\right)=\frac{1}{2}W^{T}\left(t_{b}\right)\Gamma^{-1}W\left(t_{b}\right), \end{array}$$

and the invariant takes the form

(28) $$\begin{array}{ccc} I_{0}\left(t_{b}\right) & = & \frac{1}{\omega_{1}\left(t_{b}\right)}\left[\frac{p_{1}^{2}}{2}+\frac{1}{2}\omega_{1}^{2}\left(t_{b}\right){\left(q_{1}-\frac{F_{1}\left(t_{b}\right)}{\omega_{1}^{2}\left(t_{b}\right)}\right)}^{\;\;2}\right]\\ & + & \frac{1}{\omega_{2}\left(t_{b}\right)}\left[\frac{p_{2}^{2}}{2}+\frac{1}{2}\omega_{2}^{2}\left(t_{b}\right){\left(q_{2}-\frac{F_{2}\left(t_{b}\right)}{\omega_{2}^{2}\left(t_{b}\right)}\right)}^{\;\;2}\right]\;. \end{array}$$

Using number operators $n_{1,2}$ for the oscillators along $q_{1}$ and $q_{2}$, the invariant is at boundary times $I_{0}\left(t_{b}\right)=n_{1}\left(t_{b}\right)+n_{2}\left(t_{b}\right)+1$. We define for simplicity the shifted invariant $I_{+}\left(t\right)=I_{0}\left(t\right)-1$, which at boundary times is a pure sum of number operators. The spectrum of $I_{+}\left(t\right)$ is constant and degenerate for all eigenvalues except zero (the ground state of the invariant). This poses a “degeneracy problem” since inverse engineering of the Hamiltonian based solely on $I_{0}\left(t\right)$ does not guarantee a one-to-one mapping between initial and final eigenstates except for the ground state. Just as an example, let us take the initial state $|\psi \left(0\right)\rangle ={|1,0\rangle }_{i}$. The driving protocol corresponding to λ=0, $b_{6}=0$, and $t_{f}=3$, gives the final linear combination $|\psi \left(t_{f}\right)\rangle =0.8238\;e^{-0.0197i}{|0,1\rangle }_{f}+0.5668\;e^{1.531i}{|1,0\rangle }_{f}$ due to the degeneracy of ${|0,1\rangle }_{f}$ and ${|1,0\rangle }_{f}$. We solve this problem in the next section making use of additional invariants to design the driving protocol. (Our notational convention here for initial (subscript *i*) and final (subscript *f*) eigenstates of the Hamiltonian is that the quantum numbers in ${|n,k\rangle }_{i,f}$ refer to the oscillators along $q_{1}$ and $q_{2}$).

## 6. Linear Invariants

To solve the degeneracy problem and get a more explicit expression for the final state, we need to introduce more invariants. The linear operators [17,18,19]

(29) $$G\left(t\right)=u_{x}\left(t\right)p_{x}-{\dot{u}}_{x}\left(t\right)x+u_{y}\left(t\right)p_{y}-{\dot{u}}_{y}\left(t\right)y+f\left(t\right)$$

are invariants for the Hamiltonian (12) provided

$$\begin{array}{ccc} {\ddot{u}}_{x}+M_{xx}\left(t\right)u_{x}\; & = & \;-M_{xy}\left(t\right)u_{y}, \end{array}$$

(30) $$\begin{array}{ccc} {\ddot{u}}_{y}+M_{yy}\left(t\right)u_{y}\; & = & \;-M_{xy}\left(t\right)u_{x}, \end{array}$$

(31) $$\begin{array}{ccc} \dot{f}\left(t\right)\; & = & \;-F_{x}\left(t\right)u_{x}-F_{y}\left(t\right)u_{y}. \end{array}$$

In a “direct problem” (where *M* and *F* are given) the functions $u_{x,y}$ are classical trajectories that depend on the quadratic part *M*, and *f* may be found from them and the forces. Note that $u_{x,y}$ may be complex, with real and imaginary parts representing independent trajectories.

The linear invariant can be written in terms of the rotation coordinates and momenta as

(32) $$\begin{array}{c} G\left(t\right)\;=\;u_{1}\left(t\right)p_{1}\;-\;u_{1r}\left(t\right)q_{1}\;+\;u_{2}\left(t\right)p_{2}\;-\;u_{2r}\left(t\right)q_{2}\;+\;f\left(t\right), \end{array}$$

where

(33) $$\left(\begin{array}{c} u_{1}\\ u_{2} \end{array}\right)=U\left(t\right)\left(\begin{array}{c} u_{x}\\ u_{y} \end{array}\right),\;\;\;\;\left(\begin{array}{c} u_{1r}\\ u_{2r} \end{array}\right)=U\left(t\right)\left(\begin{array}{c} {\dot{u}}_{x}\\ {\dot{u}}_{y} \end{array}\right).$$

Defining annihilation operators $a_{z}\left(t\right)=\sqrt{\omega_{z}\left(t\right)/2}\left(q_{z}-q_{z0}\left(t\right)\right)+ip_{z}/\sqrt{2\omega_{z}\left(t\right)}$, z=1,2, with $q_{10}\left(t\right)=F_{1}\left(t\right)/\omega_{1}^{2}\left(t\right)$, $q_{20}\left(t\right)=F_{2}\left(t\right)/\omega_{2}^{2}\left(t\right)$, the linear invariant can be expressed in terms of creation and annihilation operators, in particular, at boundary times

(34) $$\begin{array}{ccc} \;G(t_{b})\;\;\; & = & \;\;\;\;\sum_{z=1,2}\;\;a_{z}^{†}\left(t_{b}\right)\left[i\sqrt{\frac{w_{z}}{2}}u_{z}\left(t_{b}\right)\;\;-\;\;\frac{u_{zr}\left(t_{b}\right)}{\sqrt{2w_{z}}}\right]\;\;-\;u_{1r}\left(t_{b}\right)q_{10}\left(t_{b}\right)\\ \;\;\; & - & \;\;\;\;\sum_{z=1,2}\;\;a_{z}\left(t_{b}\right)\left[i\sqrt{\frac{w_{z}}{2}}u_{z}\left(t_{b}\right)\;\;+\;\;\frac{u_{zr}\left(t_{b}\right)}{\sqrt{2w_{z}}}\right]\;-\;u_{2r}\left(t_{b}\right)q_{20}\left(t_{b}\right)+f\left(t_{b}\right). \end{array}$$

Different linear invariants may be constructed by choosing specific boundary conditions for $u_{1,2}$ and $u_{1r,2r}$. In particular, the initial conditions [19]

(35) $$\begin{array}{cccc} u_{1}\left(0\right) & = & i/\sqrt{2w_{1}}, & u_{1r}\left(0\right)=-\sqrt{w_{1}/2},\\ u_{2}\left(0\right) & = & 0, & u_{2r}\left(0\right)=0,\\ f(0) & = & -\sqrt{w_{1}/2}\;q_{10}\left(0\right), & \end{array}$$

define an invariant which is initially $G\left(0\right)=a_{1}\left(0\right)$.

Instead, the initial conditions (the prime distinguishes them from the ones in Equation (35))

(36) $$\begin{array}{cccc} u_{1}^{\prime }\left(0\right) & = & 0, & u_{1r}^{\prime }\left(0\right)=0,\\ u_{2}^{\prime }\left(0\right) & = & i/\sqrt{2w_{2}}, & u_{2r}^{\prime }\left(0\right)=-\sqrt{w_{2}/2},\\ f^{\prime }\left(0\right) & = & -\sqrt{w_{2}/2}\;q_{20}\left(0\right), & \end{array}$$

define a different invariant which at time zero is $G^{\prime }\left(0\right)=a_{2}\left(0\right)$.

Now we may construct corresponding quadratic invariants to form initial number operators. In fact, $I_{+}\left(0\right)=G^{†}\left(0\right)G\left(0\right)+G^{\prime }{}^{†}\left(0\right)G^{\prime }\left(0\right)=a_{1}^{†}\left(0\right)a_{1}\left(0\right)+a_{2}^{†}\left(0\right)a_{2}\left(0\right)$. From Equation (28), the corresponding final invariant is $I_{+}\left(t_{f}\right)=G^{†}\left(t_{f}\right)G\left(t_{f}\right)+G^{\prime }{}^{†}\left(t_{f}\right)G^{\prime }\left(t_{f}\right)=a_{1}^{†}\left(t_{f}\right)a_{1}\left(t_{f}\right)+a_{2}^{†}\left(t_{f}\right)a_{2}\left(t_{f}\right)$, which means the “final” linear invariants $G(t_{f})$ and $G^{\prime }\left(t_{f}\right)$ have no independent terms, or terms that depend only on $a_{1}^{†}\left(t_{f}\right)$ or $a_{2}^{†}\left(t_{f}\right)$. A consequence is, see Appendix A, that the linear invariants at $t_{f}$ are given by combinations of the form

(37) $$\begin{array}{ccc} G(t_{f})\;\; & = & \;\;c_{1}a_{1}\left(t_{f}\right)+c_{2}a_{2}\left(t_{f}\right),\\ G^{\prime }\left(t_{f}\right)\;\; & = & \;\;c_{1}^{\prime }a_{1}\left(t_{f}\right)+c_{2}^{\prime }a_{2}\left(t_{f}\right), \end{array}$$

where, using Equation (34),

(38) $$\begin{array}{ccc} \;\;c_{1}\;\; & = & \;\;-i\sqrt{2w_{1}}u_{1}\left(t_{f}\right),\;\;c_{2}=-i\sqrt{2w_{2}}u_{2}\left(t_{f}\right),\\ \;\;c_{1}^{\prime }\;\; & = & \;\;-i\sqrt{2w_{1}}u_{1}^{\prime }\left(t_{f}\right),\;\;c_{2}^{\prime }=-i\sqrt{2w_{2}}u_{2}^{\prime }\left(t_{f}\right). \end{array}$$

The coefficients satisfy the following relations, see Equation (A3),

(39) $$c_{1}c_{1}^{*}+c_{1}^{\prime }c_{1}^{\prime }{}^{*}=1,\;\;c_{2}c_{2}^{*}+c_{2}^{\prime }c_{2}^{\prime }{}^{*}=1,\;\;c_{1}^{*}c_{2}+c_{1}^{\prime }{}^{*}c_{2}^{\prime }=0.$$

Considering the normalization condition at the final time, we also have the additional relations [7]

(40) $$c_{1}c_{1}^{*}+c_{2}c_{2}^{*}=1,\;\;\;c_{1}^{\prime }c_{1}^{\prime }{}^{*}+c_{2}^{\prime }c_{2}^{\prime }{}^{*}=1.$$

Finally, combining Equations (38) and (39), we have

(41) $$\begin{array}{c} c_{1}c_{1}^{*}=c_{2}^{\prime }c_{2}^{\prime }{}^{*},\;\;c_{2}c_{2}^{*}=c_{1}^{\prime }c_{1}^{\prime }{}^{*}. \end{array}$$

**Figure 3:**
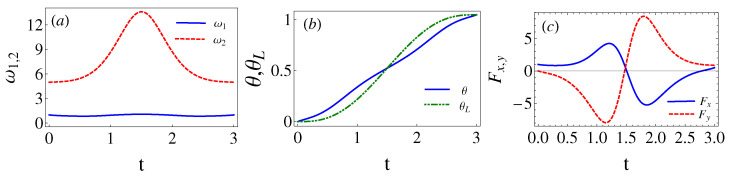
(Color online). Driving protocol for $\theta_{f}=\pi /3$, $b_{6}=24.35$, λ=34.84, $t_{f}=3$, $w_{1}=1$, and $w_{2}=5$. In (**a**), $\omega_{1}$ (blue solid line) and $\omega_{2}$ (red dashed line). In (**b**), θ (blue solid line) and $\theta_{L}$ (green dashed line). In (**c**), $F_{x}$ (blue solid line), and $F_{y}$ (red dashed line).

**Figure 4:**
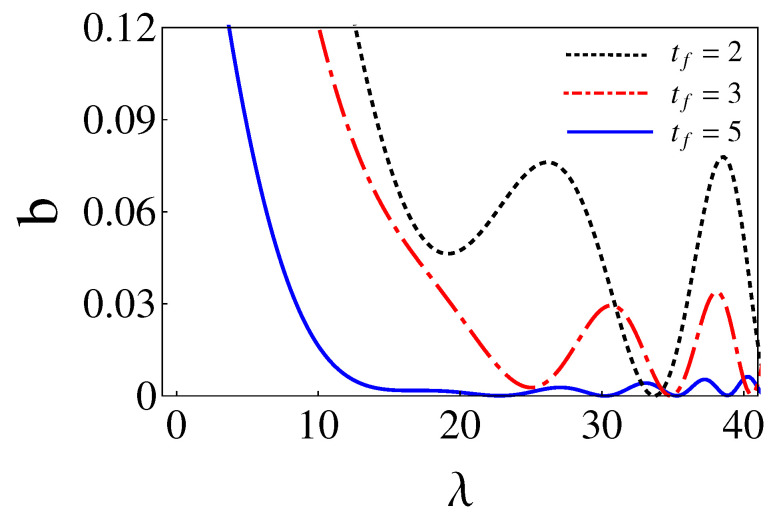
(Color online). *b* versus λ for different $t_{f}$. Parameters: $\theta_{f}=\pi /3$, $w_{1}=1$, and $w_{2}=5$. The values of $b_{6}$ are in Table 1. They are chosen to minimize *b*.

It is useful to define

(42) $$b=c_{2}c_{2}^{*}=c_{1}^{\prime }c_{1}^{\prime }{}^{*},$$

since the zeros of *b*, which can be found for special λ and $b_{6}$ values, see some examples in Table 1, achieve a remarkable simplification, namely,

(43) $$\begin{array}{ccc} G(0) & = & a_{1}\left(0\right)\to G\left(t_{f}\right){|}_{b=0}=e^{i\phi_{1}}a_{1}\left(t_{f}\right),\\ G^{\prime }\left(0\right) & = & a_{2}\left(0\right)\to G^{\prime }\left(t_{f}\right){|}_{b=0}=e^{i\phi_{2}^{\prime }}a_{2}\left(t_{f}\right), \end{array}$$

where the non-zero coefficients have unit modulus, according to Equation (40), and the phases are found from the final conditions of the trajectories, see Equation (38). These special values of λ and $b_{6}$ are found here by running classical trajectories in the inertial (xy) lab frame. The initial conditions are found from Equation (35). Fixing $b_{6}$ and sweeping on λ values, $b=|c_{2}{|}^{2}$ is calculated for a given $\left\{b_{6},\lambda \right\}$ pair from the final conditions using Equation (38). (Alternatively Equation (36) could also be used for the initial conditions with *b* computed as $|c_{1}^{\prime }{|}^{2}$ from the final conditions.) In Figure 3, corresponding to Figure 1, the eigenfrequency $\omega_{1,2}$, the coordinate rotation angle θ, the polar angle for the center of the trap $\theta_{L}$, and the force $F_{x,y}$ are plotted for the parameters $\theta_{f}=\pi /3$, $b_{6}=24.35$, and λ=34.84, which make b=0. As shown in Figure 4, for a chosen $b_{6}$ there may be several λ that make b=0 or extremely small (say $10^{-9}$). In general, we pick up the smallest λ as larger values imply tighter traps [7]. $b_{6}$ may be as well optimized by looking for the smallest *b* values found this way, for example, see Table 1. The search could be done using different optimization subroutines but we are only interested in a proof of principle here. Note also in Figure 4 that *b* flattens for larger and larger times, indeed, for very large $t_{f}$ (∼30 or larger for the parameters in the examples) the process becomes adiabatic and b=0 for any λ.

Since the ground state of the invariant is not degenerate, the initial state ${|0,0\rangle }_{i}$ will lead to ${|0,0\rangle }_{f}$ up to a phase factor. In a given basis the final state that dynamically evolves from ${|0,0\rangle }_{i}$ will generally be affected by a Lewis–Riesenfeld phase, i.e., it will be $e^{\Phi_{00}}{|0,0\rangle }_{f}$ with a $\Phi_{00}$ that depends on the specific transient protocol, $\Phi_{00}=\int_{0}^{t_{f}}dt^{\prime }\langle \Psi_{g}\left(t^{\prime }\right)\left|\left[i\frac{\partial }{\partial t^{\prime }}-H\left(t^{\prime }\right)\right]\right|\Psi_{g}\left(t^{\prime }\right)\rangle$, where $\Psi_{g}\left(t\right)$ is the instantaneous ground state of the invariant $I_{0}\left(t\right)$. For a given protocol this turns out to be a “global phase” common to all states, see Equation (45) below, so we may ignore it, as in [7], which amounts to redefining the final state basis absorbing the common phase. Such a phase would be relevant though in interferometric processes with different time-dependent protocols applied to the wavefunction branches [20,21]. An arbitrary initial eigenstate of H(0) may be written from the ground state as $|\psi \left(0\right)\rangle ={|n,k\rangle }_{i}=I_{n,k}\left(0\right){|0,0\rangle }_{i}$, where

(44) $$\begin{array}{c} I_{n,k}\left(0\right)=\frac{{\left[G^{†}\left(0\right)\right]}^{n}{\left[G^{\prime †}\left(0\right)\right]}^{k}}{\sqrt{n!k!}}=\frac{{\left[a_{1}^{†}\left(0\right)\right]}^{n}{\left[a_{2}^{†}\left(0\right)\right]}^{k}}{\sqrt{n!k!}}, \end{array}$$

where we have used the boundary conditions (35) and (36) of the auxiliary trajectories to define G(0) and $G^{\prime }\left(0\right)$. An invariant $I_{n,k}\left(t\right)$ is constructed with G(0)→G(t) and $G^{\prime }\left(0\right)\to G^{\prime }\left(t\right)$ using the corresponding trajectories. An invariant acting on a solution of the Schrödinger equation is also a solution. In particular $I_{n,k}\left(0\right){|0,0\rangle }_{i}$ will evolve dynamically to $I_{n,k}\left(t_{f}\right){|0,0\rangle }_{f}$,

(45) $$\begin{array}{c} |\psi \left(t_{f}\right)\rangle \;\;=\;I_{n,k}\left(t_{f}\right){|0,0\rangle }_{f}\\ =\frac{{\left[G^{†}\left(t_{f}\right)\right]}^{n}{\left[G^{\prime }{}^{†}\left(t_{f}\right)\right]}^{k}}{\sqrt{n!k!}}{|0,0\rangle }_{f}\\ =\frac{{\left[c_{1}^{*}a_{1}^{†}\left(t_{f}\right)\;+\;c_{2}^{*}a_{2}^{†}\left(t_{f}\right)\right]}^{n}{\left[c_{1}^{\prime *}a_{1}^{†}\left(t_{f}\right)\;+\;c_{2}^{\prime *}a_{2}^{†}\left(t_{f}\right)\right]}^{k}}{\sqrt{n!k!}}\;{|0,0\rangle }_{f}. \end{array}$$

For the special parameters λ and $b_{6}$ such that b=0, $c_{1}=e^{i\phi_{1}}$, and $c_{2}^{\prime }=e^{i\phi_{2}^{\prime }}$ while the other coefficients vanish, $c_{2}=c_{1}^{\prime }=0$. Thus, Equation (45) can be simplified as

(46) $$\begin{array}{ccc} |\psi (t_{f})\rangle & = & \frac{{\left[c_{1}^{*}a_{1}^{†}\left(t_{f}\right)\right]}^{n}{\left[c_{2}^{\prime }{}^{*}a_{2}^{†}\left(t_{f}\right)\right]}^{k}}{\sqrt{n!k!}}{|0,0\rangle }_{f}\\ & = & e^{-i(n\phi_{1}+k\phi_{2}^{\prime })}{|n,k\rangle }_{f}, \end{array}$$

which realizes a one-to-one mapping. These special parameters λ and $b_{6}$ can be found in Table 1 and Figure 4.

Finally, let us summarize the steps to design the protocol in practice: Boundary conditions at initial and final times $\left(t_{b}=0,t_{f}\right)$ are set first for the quadratic and linear parts of the potential, $M(t_{b})$, and $F(t_{b})$. The auxiliary functions R(t) and L(t) are interpolated according to expressions in Equations (22) and (23), see their physical meaning below Equation (8). Using the form of R(t) in Equation (22), M(t) in Equation (6) can be solved with two unknown parameters λ and $b_{6}$. For given λ and $b_{6}$, the system Equation (30) can be solved with the initial conditions in Equations (35) and (36), and the coefficients $c_{1,2}$, $c_{1,2}^{\prime }$ are found from Equation (38). The final state for some initial eigenstate of the Hamiltonian is given in Equation (45) in terms of these coefficients. To go from $|\psi_{n,k}\left(0\right)\rangle$ to $|\psi_{n,k}\left(t_{f}\right)\rangle$ up to a phase, avoiding the degeneracy problem, b=0 must be satisfied, see Equation (42), so that λ and $b_{6}$ cannot be arbitrary. They can be chosen with different optimization subroutines, in particular, we choose to minimize *b* and λ, to avoid traps that are too tight. Once $b_{6}$ and λ are fixed so that b=0, the time-dependent potential that defines the protocol is defined by the quadratic part M(t), and by the linear part F(t), which is found using Equation (7).

## 7. Conclusions and Discussion

In this paper, we have designed time-dependent driving protocols to map the eigenstates of an initial harmonic trap into corresponding eigenstates of a final trap, which is translated and rotated with respect to the initial one. The processing time for the one-to-one mapping can be chosen beforehand, in particular, the process may be faster than the adiabatic one, and the eigenstates whose combination form the initial state need not be known, in other words, the protocol is initial-state independent. Quadratic and linear invariants are used for inverse engineering the driving harmonic potential. The linear ones are needed to solve the “degeneracy problem” posed by the quadratic invariant proposed in [8].

**Figure 5:**
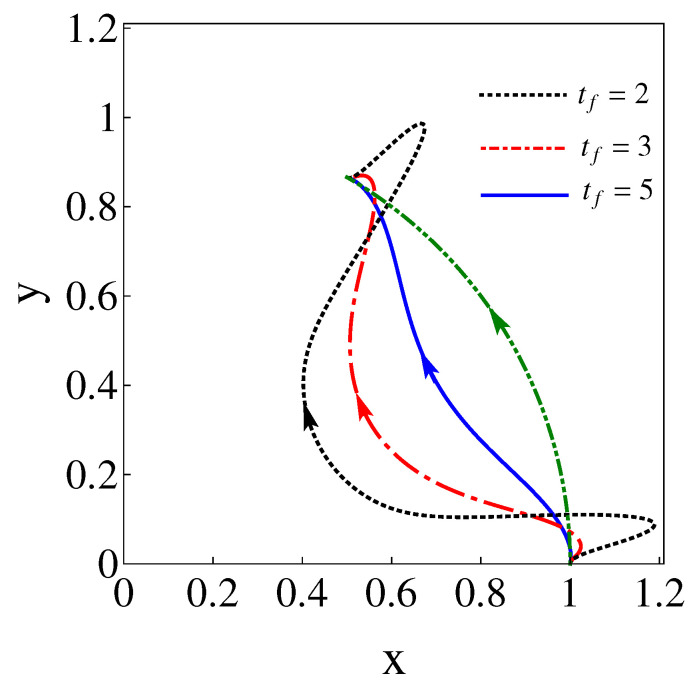
(Color online). Trajectories of the trap center for different total times ($t_{f}=2,3,5$) to drive the states along the arc. The values for $b_{6}$ and λ that make b=0, assuring a one-to-one dynamical mapping between initial and final eigenstates are given in Table 1. Initial and final traps are set as in Figure 1, with the long axis in radial directions from the origin. $w_{1}=1$, $w_{2}=5$.

Physical realizations may be performed for neutral atoms with optical traps or trapped ions with 2D Paul traps. Shorter times may imply larger and possibly imaginary potential eigenfrequencies, as well as trap trajectories that exceed a given spatial domain, see as an illustration of this point trap-center trajectories found for $t_{f}=2$, $t_{f}=3$, and $t_{f}=5$ in Figure 5. Note, in particular, that shorter times imply larger deviations of the wavepacket trajectory (green-line arch) with respect to the trap trajectory (rest of lines) and, therefore, larger potential energies. These aspects set in practice the limits for the applicability of the approach, which will depend on each specific setting and its technical constraints regarding allowed spatial domains, frequencies, energies, and time resolutions.

While our method uses invariant-based inverse engineering, this is just one among other shortcut-to-adiabaticity approaches [16] that may be worth exploring, for example, transitionless quantum driving [22], fast-forward approach [23], or time-rescaling methods [24,25]. We also note that trap rotations of charged particles have been studied with shortcut methods [26,27,28]. However, the present approach is also applicable to neutral particles. There are also many examples that show the usefulness of combining STA with optimal control theory [16]. Such a combined approach would be interesting in particular to find optimal $b_{6}$ and λ parameters.

Finally, note that the Hamiltonian forms used are also valid for other systems such as two coupled oscillators on a line, coupled superconducting qubits [29,30,31,32,33], and optomechanical oscillators [34,35,36].

## Figures and Tables

**Table 1:** Examples of parameters $b_{6}$ and λ that realize b=0 for $\theta_{f}=\pi /3$, $w_{1}=1$, $w_{2}=5$.

$b_{6}$	λ	$t_{f}$
23.55	33.694	2
24.35	34.846	3
21.10	30.221	5

## Data Availability

Not applicable.

## Appendix A. Final Invariant

For the invariant $G\left(0\right)=a_{1}\left(0\right)$ and $G^{\prime }\left(0\right)=a_{2}\left(0\right)$, the final invariants may generally be of the form

(A1) $$\begin{array}{ccc} G(t_{f}) & = & c_{1}a_{1}\left(t_{f}\right)+c_{2}a_{2}\left(t_{f}\right)+c_{3}a_{1}^{†}\left(t_{f}\right)+c_{4}a_{2}^{†}\left(t_{f}\right)+A,\\ G^{\prime }\left(t_{f}\right) & = & c_{1}^{\prime }a_{1}\left(t_{f}\right)+c_{2}^{\prime }a_{2}\left(t_{f}\right)+c_{3}^{\prime }a_{1}^{†}\left(t_{f}\right)+c_{4}^{\prime }a_{2}^{†}\left(t_{f}\right)+A^{\prime }\;, \end{array}$$

so

(A2) $$\begin{array}{ccc} I_{+}\left(t_{f}\right) & = & G^{†}\left(t_{f}\right)G\left(t_{f}\right)+G^{\prime }{}^{†}\left(t_{f}\right)G^{\prime }\left(t_{f}\right)\\ & = & a_{1}^{†}a_{1}\left(c_{1}^{*}c_{1}+c_{3}^{*}c_{3}+c_{1}^{\prime }{}^{*}c_{1}^{\prime }+c_{3}^{\prime }{}^{*}c_{3}^{\prime }\right)+a_{2}^{†}a_{2}\left(c_{2}^{*}c_{2}+c_{4}^{*}c_{4}+c_{2}^{\prime }{}^{*}c_{2}^{\prime }+c_{4}^{\prime }{}^{*}c_{4}^{\prime }\right)\\ & + & a_{1}^{†}a_{2}\left(c_{1}^{*}c_{2}+c_{4}^{*}c_{3}+c_{1}^{\prime }{}^{*}c_{2}^{\prime }+c_{4}^{\prime }{}^{*}c_{3}^{\prime }\right)+a_{2}^{†}a_{1}\left(c_{2}^{*}c_{1}+c_{3}^{*}c_{4}+c_{2}^{\prime }{}^{*}c_{1}^{\prime }+c_{3}^{\prime }{}^{*}c_{4}^{\prime }\right)\\ & + & a_{1}^{†}a_{2}^{†}\left(c_{1}^{*}c_{4}+c_{2}^{*}c_{3}+c_{1}^{\prime }{}^{*}c_{4}^{\prime }+c_{2}^{\prime }{}^{*}c_{3}^{\prime }\right)+a_{1}a_{2}\left(c_{4}^{*}c_{1}+c_{3}^{*}c_{2}+c_{4}^{\prime }{}^{*}c_{1}^{\prime }+c_{3}^{\prime }{}^{*}c_{2}^{\prime }\right)\\ & + & a_{1}^{†}a_{1}^{†}\left(c_{1}^{*}c_{3}+c_{1}^{\prime }{}^{*}c_{3}^{\prime }\right)+a_{2}^{†}a_{2}^{†}\left(c_{2}^{*}c_{4}+c_{2}^{\prime }{}^{*}c_{4}^{\prime }\right)+a_{1}a_{1}\left(c_{3}^{*}c_{1}+c_{3}^{\prime }{}^{*}c_{1}^{\prime }\right)+a_{2}a_{2}\left(c_{4}^{*}c_{2}+c_{4}^{\prime }{}^{*}c_{2}^{\prime }\right)\\ & + & a_{1}^{†}\left(c_{1}^{*}A+c_{3}A^{*}+c_{1}^{\prime }{}^{*}A^{\prime }+c_{3}^{\prime }A^{\prime }{}^{*}\right)+a_{2}^{†}\left(c_{2}^{*}A+c_{4}A^{*}+c_{2}^{\prime }{}^{*}A^{\prime }+c_{4}^{\prime }A^{\prime }{}^{*}\right)\\ & + & a_{1}\left(c_{3}^{*}A+c_{1}A^{*}+c_{3}^{\prime }{}^{*}A^{\prime }+c_{1}^{\prime }A^{\prime }{}^{*}\right)+a_{2}\left(c_{4}^{*}A+c_{2}A^{*}+c_{4}^{\prime }{}^{*}A^{\prime }+c_{2}^{\prime }A^{\prime }{}^{*}\right)\\ & + & A^{*}A+A^{\prime }{}^{*}A^{\prime }+c_{3}^{*}c_{3}+c_{3}^{\prime }{}^{*}c_{3}^{\prime }+c_{4}^{*}c_{4}+c_{4}^{\prime }{}^{*}c_{4}^{\prime }, \end{array}$$

From Equation (28), $I_{+}\left(t_{f}\right)=G_{1}^{†}\left(t_{f}\right)G_{1}\left(t_{f}\right)+G_{2}^{†}\left(t_{f}\right)G_{2}\left(t_{f}\right)=a_{1}^{†}\left(t_{f}\right)a_{1}\left(t_{f}\right)+a_{2}^{†}\left(t_{f}\right)$$a_{2}\left(t_{f}\right)$. This imposes that all the coefficients in Equation (A2) for quadratic and linear terms will be 0, except the coefficients of $a_{1}^{†}\left(t_{f}\right)a_{1}\left(t_{f}\right)$ and $a_{2}^{†}\left(t_{f}\right)a_{2}\left(t_{f}\right)$. As well, the independent term should be zero,

$$A^{*}A+A^{\prime }{}^{*}A^{\prime }+c_{3}^{*}c_{3}+c_{3}^{\prime }{}^{*}c_{3}^{\prime }+c_{4}^{*}c_{4}+c_{4}^{\prime }{}^{*}c_{4}^{\prime }=0,$$

which means that

$$A=A^{\prime }=c_{3}=c_{3}^{\prime }=c_{4}=c_{4}^{\prime }=0.$$

Using this result, we get from the quadratic terms the relations

(A3) $$c_{1}^{*}c_{1}+c_{1}^{\prime }{}^{*}c_{1}^{\prime }=1,\;\;c_{2}^{*}c_{2}+c_{2}^{\prime }{}^{*}c_{2}^{\prime }=1,\;\;c_{1}^{*}c_{2}+c_{1}^{\prime }{}^{*}c_{2}^{\prime }=0.$$

Thus the final invariants will have in principle the forms

(A4) $$\begin{array}{ccc} G(t_{f}) & = & c_{1}a_{1}\left(t_{f}\right)+c_{2}a_{2}\left(t_{f}\right),\\ G^{\prime }\left(t_{f}\right) & = & c_{1}^{\prime }a_{1}\left(t_{f}\right)+c_{2}^{\prime }a_{2}\left(t_{f}\right). \end{array}$$

which are further simplified when b=0, see Equation (43).
